# Immune checkpoint inhibitor-associated hepatotoxicity and ileitis: a diagnostic challenge in a patient on long-term nivolumab therapy

**DOI:** 10.1093/omcr/omag053

**Published:** 2026-05-10

**Authors:** Farah Shahzad, Muhammad Shahzad, Maryam Tariq, Mushayyada Fatima, Pawan Kumar Thada, Muhammad Uzair Khan Niazi, Abdul Wahab, Usman Shahzad

**Affiliations:** Department of Medicine, Khyber Medical College, Rd No. 2, Rahat Abad, Peshawar 25120, Pakistan; Department of Medicine, Foundation University Medical College, Defence Ave, Islamabad 44000, Pakistan; Department of Medicine, Aziz Fatimah Medical and Dental College, Canal Expy, Faisalabad 38000, Pakistan; Department of Clinical and Radiation Oncology, Shaukat Khanum Memorial Cancer Hospital, Phase 5, Peshawar 25000, Pakistan; Department of Medicine, Sotang Primary Hospital, Solukhumbu 56000, Nepal; Department of Anatomy, Foundation University Medical College, Defence Ave, Islamabad 44000, Pakistan; Department of Neurology, Fauji Foundation Hospital, Jhelum road, Rawalpindi 46000, Pakistan; Department of Medicine, Parkview Regional Medical Center, Fort Wayne, IN 46845, United States

**Keywords:** oncology

## Abstract

**Introduction:**

Immune checkpoint inhibitors (ICIs) improve cancer outcomes but may cause immune-related adverse events (irAEs). Nivolumab, an anti-PD-1 agent, is associated with hepatotoxicity and gastrointestinal toxicity.

**Case Presentation:**

A 33-year-old American male with anaplastic large cell lymphoma of the lung, maintained on nivolumab monotherapy for five years, presented in late 2024 with fever (102.8°F), tachycardia (130 bpm), fatigue, nausea, vomiting, and abdominal discomfort. Laboratory tests revealed mild transaminitis; infectious workup was negative. CT imaging showed mild hepatic steatosis, distal ileitis, and mesenteric lymphadenopathy. Suspected nivolumab toxicity led to permanent discontinuation of therapy and initiation of high-dose corticosteroids, resulting in rapid improvement. He was discharged on day four with a steroid taper and TMP-SMX prophylaxis but was lost to follow-up.

**Conclusion:**

This case emphasizes the potential for delayed nivolumab-induced hepatotoxicity and ileitis, underscoring the importance of early recognition and prompt management of late-onset irAEs.

## Introduction

Immune checkpoint inhibitors (ICIs) have transformed cancer therapy, significantly improving outcomes in a wide range of malignancies [1]. By May 2023, a total of eleven ICIs had been approved by the US Food and Drug Administration, demonstrating survival benefits across multiple tumor types [2]. These include cytotoxic T-lymphocyte–associated antigen 4 (CTLA-4) inhibitors, such as ipilimumab, and programmed cell death protein 1 (PD-1) inhibitors, including pembrolizumab, nivolumab, and cemiplimab, as well as programmed cell death ligand 1 (PD-L1) inhibitors, such as atezolizumab and durvalumab [2].

With growing use of ICIs in clinical practice, immune-related adverse events (irAEs) are increasingly recognized [3]. Reported irAEs range from mild (rash, diarrhea, vitiligo) to life-threatening conditions, such as type 1 diabetes, myasthenia gravis, or nephritis [3]. The likelihood of irAEs affecting any organ is estimated as high as 82%–86% with PD-1, PD-L1, and CTLA-4 blockade [3].

Immune-mediated hepatotoxicity (IMH) typically manifests as asymptomatic elevation of aminotransferases, but severe cases may present with jaundice, malaise, or liver failure requiring transplantation [4]. First-line management involves glucocorticoids, with additional immunosuppressive therapy reserved for refractory cases [6].

We present a case of a 33-year-old male with anaplastic large cell lymphoma (ALCL) treated with prolonged nivolumab, who developed immune-mediated hepatotoxicity and ileitis. This case highlights the importance of recognizing delayed irAEs in patients on long-term immunotherapy.

## Case presentation

A 33-year-old male was diagnosed six years earlier with anaplastic large cell lymphoma (ALCL) involving the left upper lung with chest wall invasion, a mass anterior to the left hepatic lobe, and supraclavicular lymphadenopathy. First-line treatment with gemcitabine and cisplatin achieved a partial response but left residual disease. He subsequently received peripheral-type benzodiazepine receptor–directed therapy with limited benefit. Salvage nivolumab combined with brentuximab vedotin stabilized disease for approximately one year, after which brentuximab was discontinued and nivolumab monotherapy continued for about five years. During this period, the patient remained in partial remission but experienced repeated admissions for pneumonia, sepsis, weight loss, and debility. Although nivolumab was used off-label, continuation was justified due to limited standard options and emerging evidence supporting PD-1 blockade in T-cell lymphomas.

His past medical history included deep vein thrombosis of the left upper extremity and multiple episodes of community-acquired pneumonia. He had no diabetes, hypertension, or autoimmune disease.

In late 2024, he presented to the emergency department with fever (102.8°F), tachycardia (130 bpm), fatigue, poor appetite, intermittent nausea, vomiting, headaches, body aches, cough, and diffuse abdominal discomfort. He denied diarrhea, constipation, chest pain, dyspnea, urinary symptoms, or weight change. On examination, he appeared febrile, tachycardic, and mildly ill. Conjunctiva were pink, sclera anicteric, and he reported mild ocular itching. Oral mucosa was moist, the neck was supple without lymphadenopathy, and chest examination revealed symmetrical expansion with clear breath sounds. Cardiac assessment showed normal S1 and S2 without murmurs. The abdomen was soft, non-tender, and demonstrated active bowel sounds. Extremities were without edema, and pulses were full and equal. A chest port was present.

Laboratory work revealed mild transaminitis (details in Table 1). Serologies for CMV, HIV, EBV, influenza, and SARS-CoV-2 were negative, and urine analysis was unremarkable. Chest X-ray demonstrated elevation of the left hemidiaphragm with discoid and linear opacities, likely atelectasis, without effusion or pneumothorax. CT of the head was normal. CT of the chest revealed scarring and bronchiectasis in the medial left lung base with no adenopathy. Contrast-enhanced CT of the abdomen and pelvis showed mild hepatic steatosis and inflammatory changes in the distal ileum and cecum with reactive mesenteric lymphadenopathy, consistent with immune-related toxicity. No obstruction, ascites, or appendicitis was identified. Nasal swab PCR was negative for *Staphylococcus aureus*, including MRSA.

**Table 1:** Serial laboratory investigations during hospital admission.

Investigation	Normal Range (US)	Day of Presentation	Day 1 of Admission	Day 2 of Admission
CBC				
WBC (×10^9^/l)	4.5–11.0	18.6	14.3	13.3
RBC (×10^12^/l)	Male: 4.7–6.1 Female: 4.2–5.4	4.2	3.6	3.54
Hematocrit (%)	Male: 40–52 Female: 36–48	37.8	32.3	31.4
Hemoglobin (g/dl)	Male: 13.5–17.5 Female: 12–15.5	13.4	11.5	11.1
Differential Count – Relative (%)				
Neutrophils	40–70	84.8	82.2	–
Lymphocytes	20–45	4.1	5.7	–
Monocytes	2–10	8.9	9.5	–
Eosinophils	1–6	0.3	1.3	–
Basophils	0–1	0.6	0.1	–
Immature Grans	<0.4	1.3	1.2	–
Differential Count – Absolute (×10^9^/l)				
Neutrophils	1.5–7.5	15.7	11.74	–
Lymphocytes	1.0–4.0	0.76	0.81	–
Monocytes	0.2–0.8	1.66	1.35	–
Eosinophils	0.02–0.5	0.06	0.19	–
Basophils	<0.1	0.11	0.02	–
Immature Grans	<0.03	0.24	0.17	–
Electrolytes				
Sodium (mmol/l)	135–145	134	140	139
Potassium (mmol/l)	3.5–5.0	4.3	3.5	3.4
Chloride (mmol/l)	96–106	94	102	102
Renal Function				
BUN (mg/dl)	7–20	9	9	6
Creatinine (mg/dl)	0.6–1.3	1.33	1.37	1.48
Proteins				
Total Protein (g/dl)	6.0–8.3	8.1	5.9	6.0
Albumin (g/dl)	3.5–5.0	3.8	3.1	2.9
Globulin (g/dl)	2.0–3.5	4.3	2.8	3.1
A/G ratio	1.0–2.0	0.9	1.1	0.9
Bilirubin				
Total (mg/dl)	0.3–1.2	1.9	1.8	1.9
Direct (mg/dl)	0.1–0.4	0.9	1.4	1.5
Indirect (mg/dl)	0.2–0.8	1.1	0.4	0.4
Liver Enzymes				
AST (U/L)	10–40	173	119	123
ALT (U/L)	7–56	330	251	240
ALP (U/L)	44–147	342	268	248

Given the febrile and immunocompromised presentation, empiric broad-spectrum antibiotics were initiated. After infectious causes were excluded, antibiotics were discontinued, and nivolumab-related immune toxicity was suspected. Immunotherapy was permanently discontinued after multidisciplinary oncology consultation. High-dose intravenous corticosteroids were initiated, leading to resolution of fever and symptomatic improvement. Due to anticipated prolonged steroid therapy, trimethoprim-sulfamethoxazole prophylaxis was prescribed for Pneumocystis jirovecii pneumonia.

The patient became afebrile, tolerated oral intake, and was discharged on hospital day four with a steroid taper, prophylactic TMP-SMX, and close oncology follow-up. He was subsequently lost to follow-up.

## Discussion

Primary anaplastic large cell lymphoma (ALCL) of the lung is a rare tumor with nonspecific features but generally favorable prognosis when diagnosed early [5]. Nivolumab, an anti-PD-1 therapy, has shown activity in hematologic malignancies, including T-cell lymphomas [9, 10].

Immune-related adverse events (irAEs) are common with prolonged therapy, affecting skin, gastrointestinal, endocrine, hepatic, and renal systems [3, 4]. Our patient developed hepatotoxicity and ileitis while in remission on long-term nivolumab. Notably, he lacked typical irAE manifestations such as rash, diarrhea, or marked weight change, making diagnosis challenging. Infectious causes were excluded, and nivolumab toxicity was suspected based on transaminitis and imaging findings.

Nivolumab is not FDA-approved for ALCL but was used off-label as salvage therapy in this patient, justified by limited alternatives and supporting evidence [9, 10]. Hepatic injury from nivolumab usually arises within 1–3 months but can occur later. The reported incidence of clinically apparent hepatotoxicity is 1–2% [4]. Monitoring with regular labs and early recognition are critical [6]. Ileitis, another uncommon irAE, has also been reported [8].

While our case involved late-onset toxicity, other reports describe early and sometimes fatal outcomes. For example, Matsubara et al. reported steroid-resistant hepatic injury within 3 weeks of therapy [7], and fatal hepatic events following combination PD-1/CTLA-4 blockade have been reported in the literature and highlighted in clinical practice guidelines [6]. Such cases underscore the variability of onset and severity.

Early diagnosis and corticosteroid therapy can reverse most toxicities without permanent damage. Close follow-up remains essential for patients receiving prolonged immunotherapy.

## Conclusion

Clinicians should remain alert to delayed immune-related adverse events in patients on prolonged immunotherapy, as early recognition and treatment can prevent serious complications.

## Limitation

This case report has certain limitations. First, de-identified imaging could not be included due to institutional restrictions; therefore, only clinically relevant imaging findings have been described. Second, the patient was lost to follow-up, which limits the assessment of long-term outcomes. Moreover, followup labs were not available and hence counts as another limitation of the report.
